# Production and use of estimates for monitoring progress in the health sector: the case of Bangladesh

**DOI:** 10.1080/16549716.2017.1298890

**Published:** 2017-05-22

**Authors:** Karar Zunaid Ahsan, Tazeen Tahsina, Afrin Iqbal, Nazia Binte Ali, Suman Kanti Chowdhury, Tanvir M. Huda, Shams El Arifeen

**Affiliations:** ^a^ Department of Maternal and Child Health, Gillings School of Global Public Health, University of North Carolina at Chapel Hill, Chapel Hill, NC, USA; ^b^ Maternal and Child Health Division (MCHD), icddr,b, Dhaka, Bangladesh

**Keywords:** Health information system, S.D.Gs, health indicators, health data, global estimates, Bangladesh

## Abstract

**Background:** In order to support the progress towards the post-2015 development agenda for the health sector, the importance of high-quality and timely estimates has become evident both globally and at the country level.

**Objective and Methods:** Based on desk review, key informant interviews and expert panel discussions, the paper critically reviews health estimates from both the local (i.e. nationally generated information by the government and other agencies) and the global sources (which are mostly modeled or interpolated estimates developed by international organizations based on different sources of information), and assesses the country capacity and monitoring strategies to meet the increasing data demand in the coming years. Primarily, this paper provides a situation analysis of Bangladesh in terms of production and use of health estimates for monitoring progress towards the post-2015 development goals for the health sector.

**Results:** The analysis reveals that Bangladesh is data rich, particularly from household surveys and health facility assessments. Practices of data utilization also exist, with wide acceptability of survey results for informing policy, programme review and course corrections. Despite high data availability from multiple sources, the country capacity for providing regular updates of major global health estimates/indicators remains low. Major challenges also include limited human resources, capacity to generate quality data and multiplicity of data sources, where discrepancy and lack of linkages among different data sources (local sources and between local and global estimates) present emerging challenges for interpretation of the resulting estimates.

**Conclusion:** To fulfill the increased data requirement for the post-2015 era, Bangladesh needs to invest more in electronic data capture and routine health information systems. Streamlining of data sources, integration of parallel information systems into a common platform, and capacity building for data generation and analysis are recommended as priority actions for Bangladesh in the coming years. In addition to automation of routine health information systems, establishing an Indicator Reference Group for Bangladesh to analyze data; building country capacity in data quality assessment and triangulation; and feeding into global, inter-agency estimates for better reporting would address a number of mentioned challenges in the short- and long-run.

## Background

Globally, the overall progress in achieving the Millennium Development Goals (MDGs) has been encouraging, as we have witnessed an accelerated rate of reduction in maternal and child mortality rates. However, despite a 45% reduction in maternal and 53% reduction in child mortality, the progress was insufficient for us to reach MDG 4 and 5 targets globally and in some regions [1–4]. Progress was much slower than anticipated in many countries, though there were some remarkable exceptions. Moving beyond the MDG era, the world is now committed to achieving the Sustainable Development Goals (SDGs) to ensure healthy lives and promote wellbeing for all [5]. The renewed focus and commitment on maternal, newborn, and child survival have led to several global strategies and action plans: the Global Strategy For Women’s, Children’s and Adolescents’ Health, Ending Preventable Maternal Mortality (EPMM), and Every Newborn Action Plan (ENAP) [6–9]. These strategies and action plans will act as a platform to accelerate improvements in maternal and child health and targets set will help track and measure the progress for the post-2015 development agenda.

In order to support the progress towards the SDGs, the importance of high-quality and timely health estimates has become evident – both globally and at the country level [7]. However, availability and quality of women and children’s health data are very often insufficient in developing countries, making it difficult to measure progress [8]. The United Nations’ (UN) Commission on Information and Accountability for Women’s and Children’s Health (CoIA) was created in 2011 for ensuring accountability and global reporting on women’s and children’s health [10] and the Health Data Collaborative (HDC) was established in 2015 to improve measurement and accountability for the health sector and support a ‘data revolution’ to meet the demand for the post-2015 development agenda [11].

Over the last two decades, the data capturing system in the Bangladesh health sector has undergone significant reforms. A concerted effort was made for ensuring data availability to track progress towards the MDGs. Moving forward and in order to respond to the global need for reporting health data for measuring progress towards the SDGs, the Government of Bangladesh (GoB) has highlighted strengthening the health information system (HIS) as a key driver of the next health sector programme (2017–2021), building on the work done for the current programme (2011–2016) [12–16]. The Bangladesh Ministry of Health and Family Welfare (MOHFW) has approved a Monitoring and Evaluation Strategy and Action Plan (MESAP) for the health sector with a focus on strengthening the routine HIS and integration of different data sources [14]. Still, considerable effort will be needed to address the emerging demands for data in the post-2015 period [17].

## Objective

The aim of this paper is to conduct a situation analysis of Bangladesh in terms of production and use of health estimates for monitoring progress towards the post-2015 development agenda.

## Methods

This assessment is primarily based on a desk review of published and grey literature on monitoring and evaluation of the Bangladesh health sector and the sector-wide approach (SWAp) in the health sector. We also reviewed information obtained from key informant interviews and expert panel discussions during the Inter-country Conference on Measurement and Accountability for Health (MA4Health), held in Dhaka, Bangladesh, in April 2016. We have critically reviewed health estimates from both local (i.e. nationally generated information by the government and other agencies) and global sources (which are mostly modeled or interpolated estimates developed by international organizations based on different sources of information), and assessed country strategies and monitoring plans to meet the data demand for the post-2015 development agenda. Based on our analysis, we put forward recommendations to improve the production and use of estimates for monitoring progress in the Bangladesh health sector in the coming years.

## Results and discussion

### Overview of country data sources for monitoring the health sector

#### Survey data

In terms of survey data, Bangladesh is data rich – with over 35 health surveys in the last 2 decades (Table 1). Most are nationally representative population surveys, with a few health facility assessments. Nationally representative sample surveys are routinely carried out by the National Institute of Population Research and Training (NIPORT) of MOHFW and other government/non-government institutions, including the national statistics office [Bangladesh Bureau of Statistics (BBS)], with support from Development Partners (DPs). For maternal, newborn, and child health, the most widely recognized and used surveys are Bangladesh Demographic and Health Surveys (BDHS), Bangladesh Maternal Mortality Surveys (BMMS), Utilization of Essential Service Delivery (UESD) Surveys, and Multiple Indicators Cluster Surveys (MICS). These surveys usually publish their reports at intervals of 2–5 years and are considered as the most reliable sources to provide updates on a number of vital indicators of intervention coverage and population impact. NIPORT also conducts Bangladesh Health Facility Surveys (BHFSs) to assess the service provision and readiness of health facilities.

**Table 1.** Health sector surveys in Bangladesh, 1993–2014.

Survey	Rounds	Implementer	Funder
Demographic and Health Survey (DHS)	1993, 1996/97, 1999/2000, 2004, 2007, 2011, 2014	MOHFW	USAID
Utilization of Essential Service Delivery Survey (mini-DHS)	2006, 2008, 2010, 2013	MOHFW	GoB
Multiple Indicator Cluster Survey (MICS)	1993, 1995, 2006, 2009, 2012/13	BBS	UNICEF
Health and Demographic Survey (HDS)	1994, 1995, 1996, 1997, 1998, 2000, 2012	BBS	GoB
Service Delivery Survey (SDS)	1999, 2000, 2003	CIET	CIDA
Urban Health Survey (UHS)	2006, 2013	MOHFW	USAID/DfID
Maternal Mortality Survey (BMMS)	2001, 2010	MOHFW	GoB/USAID
Bangladesh Health Facility Survey (BHFS)	2009, 2011, 2014	MOHFW	GoB/WB
Child and Mother Nutrition Survey (CMNS)	1995, 2000, 2005, 2012	BBS	GoB/UNICEF

Notes: BBS – Bangladesh Bureau of Statistics, CIDA – Canadian International Development Agency, DfID – Department for International Development, MOHFW – Ministry of Health and Family Welfare, GoB – Government of Bangladesh, WB – World Bank, UNICEF – United Nations International Children’s Emergency Fund, USAID – United States Agency for International Development.

#### Data from routine HIS

Data on service contacts are collected by all health and family planning field workers and service delivery facilities (i.e. from the lowest-level Community Clinics [CCs] to tertiary-level hospitals). Community-level data of the Directorate General of Health Services (DGHS) constituting outpatient data from the CCs and Union Sub-centres come to the respective sub-district health offices where the data are aggregated and reported. Out-patient, in-patient, and emergency patient profiles from upazila- (sub-district) and district-level facilities are also aggregated at the district offices to be relayed through the Management Information System (MIS) of DGHS, which uses District Health Management Information Software System version 2 (DHIS2)-based systems. All other public hospitals send their data on patients directly to the MIS of DGHS. Service statistics of the Directorate General of Family Planning (DGFP) deals with family planning, maternal and child health (MCH), and reproductive health services. Monthly contraceptive performance and MCH reports are published from the MIS of DGFP based on aggregated data received from the field workers and facilities in the public as well as NGO (nongovernmental organizations) sectors. In addition, routine data are generated from different programmes of DGHS and DGFP on specific activities (e.g. immunization, Integrated Management of Childhood Illness [IMCI], emergency obstetric care [EmOC], etc.). While long-established data collection tools and methods are used for collecting and aggregating data at sub-district, district, and national levels, the systems are different for DGHS and DGFP, often resulting in conflicting estimates.

#### Data from other sources

The reports of the Sample Vital Registration System (SVRS) produced by B.B.S provide national and regional data on population, births and deaths, causes of death, and life expectancy. The Household Income and Expenditure Survey (HIES) of BBS also provides estimates on health expenditures at five-year intervals. Coverage Evaluation Surveys (CES) are conducted by MOHFW’s Expanded Programme on Immunization (EPI) every year and HIV sero-surveillance is conducted periodically to assess programme performance. Research institutions and academia carrying out health systems research, clinical trials, and longitudinal studies also provide useful data for health sector monitoring.

#### Data from global sources

Global data sources like the United Nations’ Inter-agency Group for Child Mortality Estimation (IGME) and the Maternal Mortality Estimation Inter-agency Group (MMEIG) are also being used for monitoring goal-level indicators of the health sector, particularly for the Annual Programme Reviews (APR) of the health sector programmes as these global estimates are often the most recent. Other notable global sources for health estimates are the Institute for Health Metrics and Evaluation’s Global Burden of Disease, World Health Organization’s (WHO) Global TB Program, and the World Bank’s World Development Indicators.

#### Use of health estimates in the government’s policies and programmes

**Figure 1.**
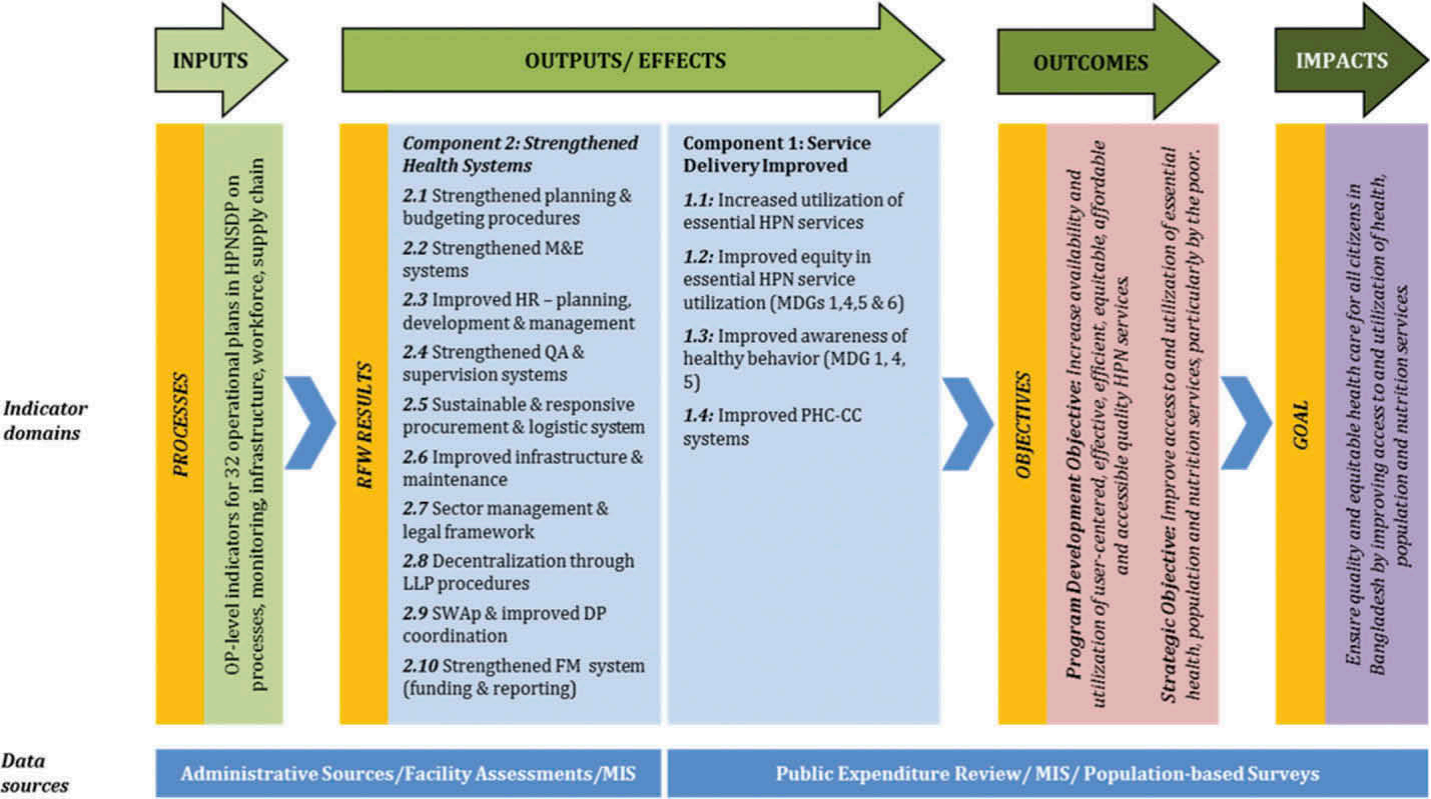
Monitoring framework for Bangladesh health sector programme.

The GoB embarked on a SWAp for the health sector in 1998. Over the years, the SWAp modality has evolved in Bangladesh as the MOHFW has learnt from implementation experiences and been refining the design of the programme. The current health sector programme, the Health, Population and Nutrition Sector Development Programme (HPNSDP) for 2011–2016, developed and implemented a monitoring framework (Figure 1) that utilizes data from both routine data sources as well as household and facility surveys described in the preceding section [14].

Other examples of using health estimates in decision making in Bangladesh are:

- Expansion of the IMCI Programme during 2002–2007, first in the districts with the highest under-five mortality rates, based on 2001 BMMS estimates;
- Extensive use of the 2010 BMMS results to inform health sector planning in Bangladesh, e.g. for selecting low-performing districts and sub-districts for the sector programme’s performance-based financing modality;
- Annual Programme Implementation Reports, heavily based on survey and routine data, have led to changes in the way coordination is managed in the sector programme; and
- The 2009 and 2014 BHFS results were used for developing action plans to address service availability and readiness issues in the health facilities.

Country use of global health estimates for evidence-based decision making, however, is a recent phenomenon for Bangladesh. In response to MOHFW’s increased demand for annual updates on goal-level indicators of the sector programme, there has been greater utilization of globally modeled estimates for policymaking and programme monitoring in Bangladesh’s health sector. In the absence of a reliable civil registration and vital statistics system (CRVS) in Bangladesh, MOHFW relies on nationally representative household surveys as the source of goal- and outcome-level estimates, which only become available every three to five years. Development assistance to MOHFW by bi- and multilateral agencies is also linked to uninterrupted availability of higher-level indicator updates and there is increasingly a move towards a results-based financing modality in the health sector. While the preference is still for data from local sources like surveys, as these are considered the most reliable, the main reason for using global, modeled estimates is unavailability of updated local data at timely intervals, e.g. annually. For the official country reports on monitoring progress towards the MDGs, GoB first used global estimates in 2010. Also in the 2015 update on Bangladesh’s progress towards the MDGs, the MOHFW used the WHO, UN and MMEIG as sources alongside country-level data sources. Global data sources like IGME and MMEIG are being increasingly used for monitoring periodic updates of the sector programmes’ goal-level indicators during the annual APRs. Also, in the GoB’s 7th Five Year Plan (FYP), which guides the overall policy directions for 2016–2021, a global estimate (maternal mortality ratio from MMEIG) has been included for the first time for monitoring FYP implementation.

### Country capacity for monitoring the health sector

MOHFW has considerably improved its capacity for monitoring and evaluation compared to previous health sector programmes [18]. To support the MOHFW in sector programme management and monitoring, a Program Management and Monitoring Unit (PMMU) was established under the Planning Wing of MOHFW. Based on lessons learned from an earlier effort, the current PMMU was formed with both GoB and external technical assistance components in the form of long-term, external specialists supported by the United States Agency for International Development (USAID) and Department for International Development (DfID). PMMU facilitated more intensive programme monitoring by the MOHFW by producing bi-annual programme implementation reports, assisting independent reviewers to conduct APR, and providing advisory services to the senior policymakers of MOHFW to address critical management and monitoring issues. The PMMU helped develop the Monitoring and Evaluation (M&E) Strategy and Action Plan, the first ever strategy on M&E for the health sector to be approved by the GoB, to focus on strengthening routine HIS and build country capacity in M&E [18].

Regular monitoring of globally recognized health indicators still remains a challenge. The WHO Department of Health Statistics and Information Systems developed the Global Reference List of 100 Core Health Indicators as a standard set of indicators prioritized by the global community to provide concise information on the health situation and trends for reporting of the post-2015 health goals [19]. Our analysis indicates that only 15 of the 100 indicators can be monitored annually through the routine HIS in Bangladesh (Table 2) [17].

**Table 2.** Global reference list of 100 core indicators by sources in Bangladesh.

	Data source							
Indicator type	Household surveys (BDHS/UESD/MICS/BMMS)	Facility surveys (BHFS)	Other/irregular surveys	Vital Registration System(SVRS)	Health Accounts/expenditure review (NHA/PER)	Sero-surveillance	Not available	Routine HIS
Health status indicators	7		1	8		4	4	3
Risk factors indicators	13		5			1	2	
Service coverage indicators	9					1	6	11
Health systems indicators	3	3	5		7		5	1
**Total**	**30**	**3**	**11**	**8**	**7**	**6**	**17**	**15**

Notes: BDHS – Bangladesh Demographic and Health Survey; UESD – Utilization of Essential Service Delivery Survey; MICS – Multiple Indicator Cluster Survey; BMMS – Bangladesh Maternal Mortality Survey; BHFS – Bangladesh Health Facility Survey; SVRS – Sample Vital Registration System; NHA – National Health Accounts; PER – Public Expenditure Reviews; HIS – Health Information System.

For the health-related SDG (Goal 3) indicators [20], Bangladesh can provide regular, valid updates at national level for only 10 indicators out of the proposed 25 indicators, with only 1 indicator being monitored using routine data (Table 3).

**Table 3.** Proposed S.D.G indicators for health (Goal 3) by local sources in Bangladesh.

	Data source							
Proposed indicators	BDHS/UESD/MICS/BMMS	BHFS	Other/irregular surveys	SVRS	NHA/PER	Sero-surveillance	Not available	Routine HIS
3.1.1 Maternal deaths per 100,000 live births	1							
3.1.2 % of births attended by skilled health personnel	1							
3.2.1 Under-five mortality rate (per 1000 live births)	1							
3.2.2 Neonatal mortality rate (per 1000 live births)	1							
3.3.1 New HIV infections per 1000 uninfected people						1		
3.3.2 Tuberculosis incidence per 1000 persons per year							1	
3.3.3 Malaria incident cases per 1000 persons per year								1
3.3.4 New hepatitis B infections per 100,000 people							1	
3.3.5 Number of people requiring interventions against neglected tropical diseases							1	
3.4.1 Mortality of cardiovascular disease, cancer, diabetes, or chronic respiratory disease				1				
3.4.2 Suicide mortality rate							1	
3.5.1 Coverage of treatment interventions for substance use disorders							1	
3.5.2 Harmful use of alcohol among aged 15 years and older within a calendar year							1	
3.6.1 Road traffic fatal injury deaths within 30 days, per 100,000 population							1	
3.7.1 % of women aged 15–49 with met need for family planning with modern methods	1							
3.7.2 Adolescent birth rate per 1000 women	1							
3.8.1 Coverage of tracer interventions (e.g. child full immunization, antiretroviral therapy, TB treatment, etc.)	1							
3.8.2 % of the population protected against impoverishing out-of-pocket health expenditure							1	
3.9.1 Mortality rate attributed to household and ambient air pollution							1	
3.9.2 Mortality rate attributed to hazardous chemicals, water and soil pollution and contamination							1	
3.a.1 Age-standardized prevalence of current tobacco use among persons aged 15 years and older			1					
3.b.1 % of the population with access to affordable medicines and vaccines on a sustainable basis		1						
3.b.2 Total net official development assistance to medical research and basic health sectors					1			
3.c.1 Health worker density and distribution								1
3.d.1 % of attributes of 13 core capacities that have been attained at a specific point in time							1	
**Total**	**7**	**1**	**1**	**1**	**1**	**1**	**11**	**2**

Notes: BDHS – Bangladesh Demographic and Health Survey; UESD – Utilization of Essential Service Delivery Survey; MICS – Multiple Indicator Cluster Survey; BMMS – Bangladesh Maternal Mortality Survey; BHFS – Bangladesh Health Facility Survey; SVRS – Sample Vital Registration System; NHA – National Health Accounts; PER – Public Expenditure Reviews; HIS – Health Information System.

### In-country data realities: gaps and challenges

#### Quality of data

Earlier studies on the health sector programme [21–25] identified unreliability of programme-related data and service statistics due to incomplete or erroneous reporting as a major reason for the lack of data use in decision-making processes at all levels [14]. A data quality assurance (DQA) system is not enforced in the routine HIS – only several vertical health programmes (e.g. EPI, EmOC) have their own DQA systems, and the MIS of DGFP conduct supervision visits (four teams each month) for validating field data reporting in selected samples. However, there is no system in place for internal DQA at regular intervals and no DQA plan exists to provide systematic oversight in monitoring the quality (including accuracy, completeness, and timeliness) of routinely collected data through the MISs.

For the survey data, quality is usually ensured through intensive quality control processes that involve teams carrying out checks and observations during the surveys. In the surveys implemented by NIPORT (BDHS, UESD, BMMS), NIPORT monitors fieldwork by using designated quality control teams. Data quality is also monitored through field check tables generated concurrently with data processing, which is particularly useful because the quality control teams are able to advise field teams of problems detected during data entry.

#### Consistency between local and global estimates

**Figure 2.**
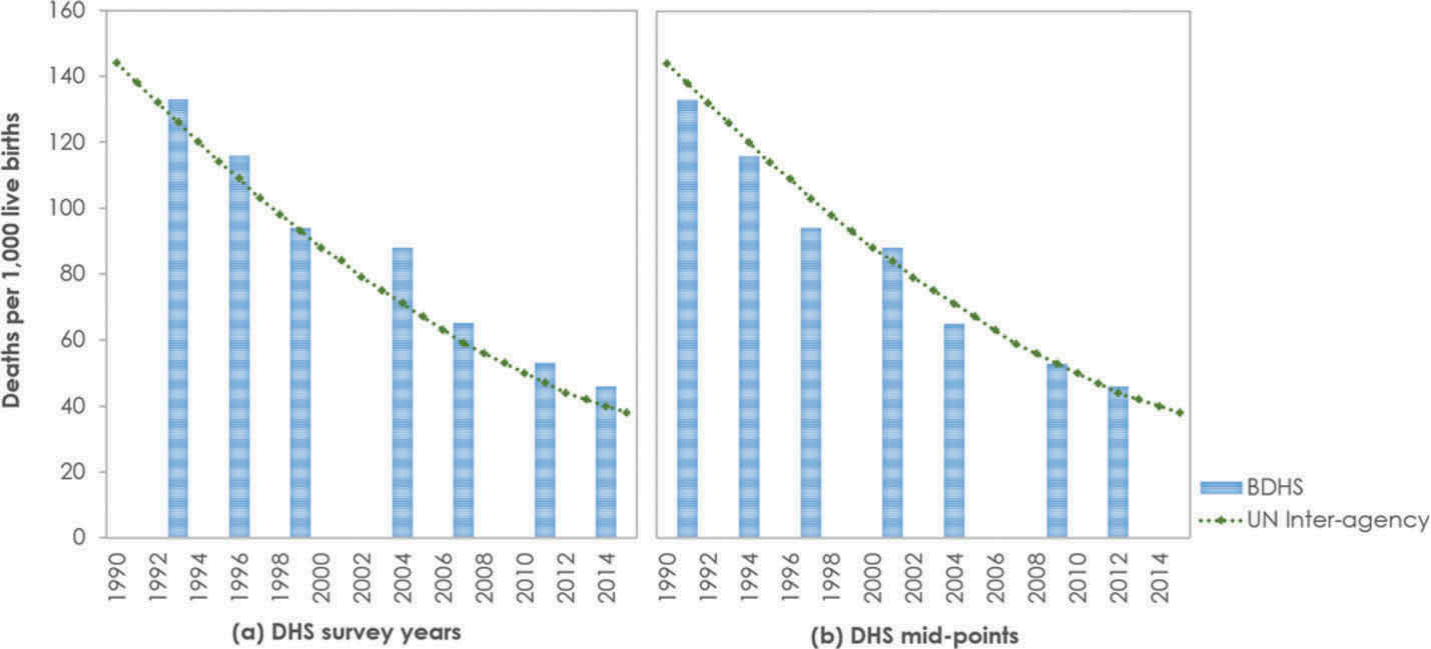
Consistency between local and global estimates for under-five mortality rate. Notes: BDHS – Bangladesh Demographic and Health Survey; UN – United Nations.

There are issues with consistency, comparability, and contextualization of estimates across different sources. Often confusion is created when both local and global estimates are available for the same indicators for overlapping time periods. Taking under-five mortality rate as an example, at first look, IGME estimates for under-five mortality appear to be systematically lower than the estimates from the Bangladesh DHS (Figure 2(a)). However, if the BDHS data points are plotted on the correct time-scale, i.e. on the mid-point of the survey reference period of the indicator rather than on the actual survey year, the estimates from local and global sources seem consistent (see Figure 2(b)).

There exist other examples where local and global estimates are not consistent – for maternal mortality ratio, the M.MEIG estimate for 2010 is 242 and for 2013 is 170 per 100,000 live births, whereas local estimates from BMMS and SVRS for the corresponding years are 194 and 197 per 100,000 live births, respectively [26–28]. Other similar examples of mismatch between local and global estimates are seen in measles immunization coverage (UESD vs. WHO/UNICEF [United Nations International Children’s Emergency Fund] joint estimates), tuberculosis (TB) case detection rate (National TB Programme vs. WHO), and cause of death (SVRS vs. IHME) [26].

Comparability of local and global estimates is also influenced by differences in the definition of the indicators across different sources. For example, ‘Global Reference List of 100 Core Indicators’ defines ‘met need for contraception’ as percentage of women of reproductive age (15–49 years) who are sexually active and who have their need for family planning satisfied with modern methods, whereas BDHS estimates unmet need for family planning based on fecund women who are not using contraception but who wish to postpone the next birth or stop childbearing altogether. Lastly, in terms of contextualization, Bangladesh needs to focus on indicators on essential newborn care as well as infant and young child feeding practices (IYCF) given the burden of neonatal mortality and undernutrition. However, the majority of these indicators are not included in the list of 100 global indicators except for exclusive breastfeeding.

#### Human resources and capacity

Monitoring progress towards the SDGs will require increased capacity to collect and analyze data. The PMMU of the Planning Wing in the MOHFW is the technical body authorized to monitor the health sector programme’s progress nationally. Despite favourable policies and significant efforts for in-service training for HR capacity development for M&E activities, the public sector continues to face the challenge of ensuring a skilled workforce for evidence-based monitoring of progress. An M&E scorecard for Bangladesh’s health sector highlighted the need for developing and implementing a multi-year capacity-building plan to address the systemic gaps and weaknesses in producing good-quality data [17]. There are also workforce shortages when it comes to filling key data management and system engineering posts in the MIS units at different levels, and training of staff involved with data entry, management, and reporting is insufficient [17]. The 2011 BHFS found that, on average, only 32% of the Health Management Information System (HMIS) forms or registers were completed in the public facilities, and less than 5% of the facility staff had received any training on completing the HMIS forms [29]. Data from the 2011 BHFS also indicated that only 15% of the district hospitals and 73% of the sub-district hospitals had their statistical assistant positon filled.

#### Multiplicity of data sources

**Figure 3.**
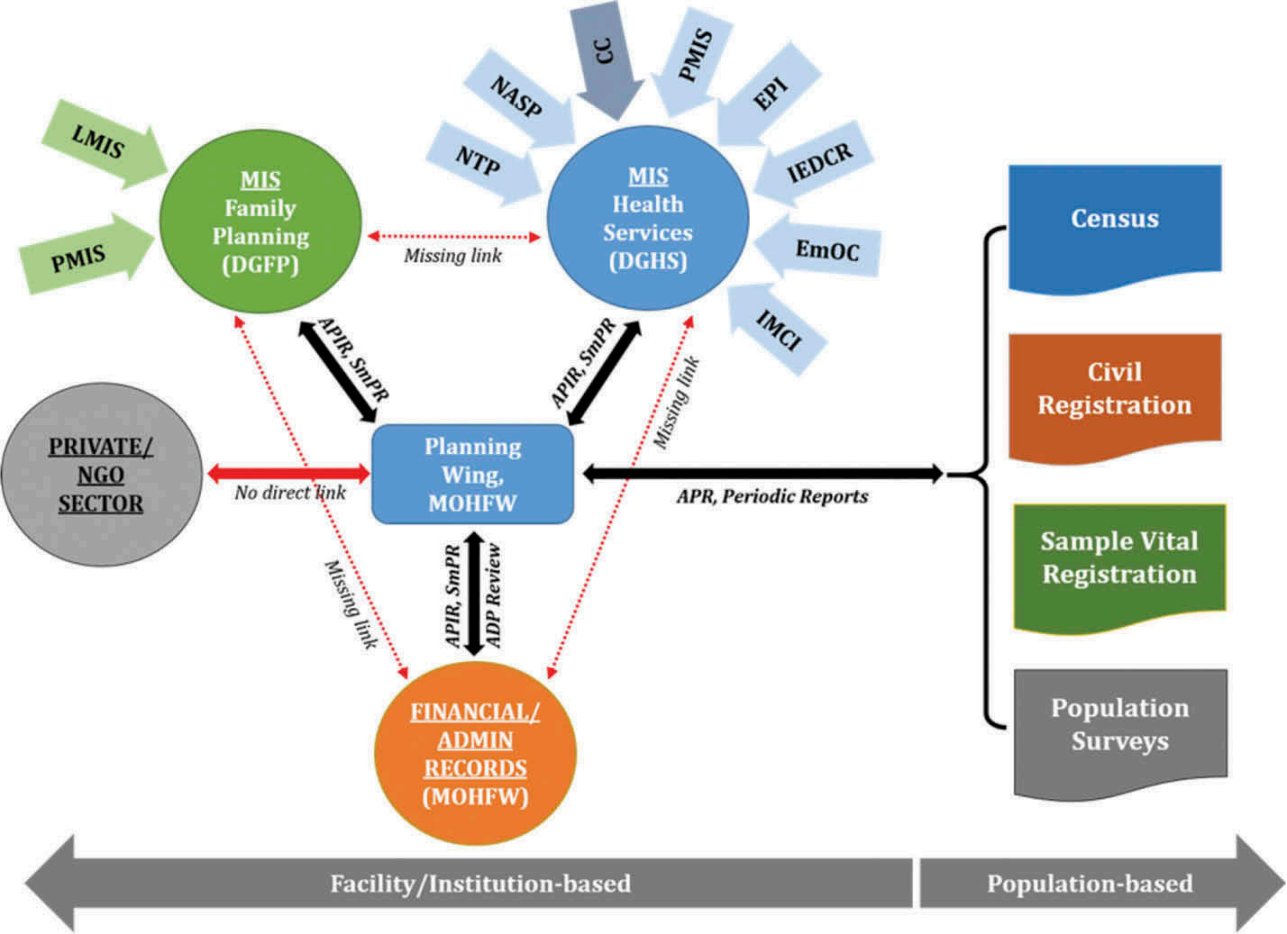
Multiplicity of local data sources for health indicators. Notes: APIR – Annual Programme Implementation Report; SmPR – Six-monthly Progress Report; ADP – Annual Development Program; APR – Annual Programme Review (of the health sector programme).

At this time, there are multiple data generation systems in the MOHFW with little or no linkages between them (Figure 3). Moreover, with the exception of a few specific programmes involving public–private partnership (e.g. National TB Programme), information from private and NGO organizations is not routinely accessed/utilized by the public sector for monitoring the sector programme.

### Recommendation and conclusion

In the coming years, the demand for data for monitoring of the SDGs will increase substantially, and a focus on equity will necessitate more disaggregated data. Worldwide interest in monitoring progress emphasizes the need for production, collection, analysis, and sharing of quality data for evidence-based decision making and investments in the health sector. As we move beyond the MDG era, the GoB is emphasizing the generation of real-time data by strengthening its routine HIS and CRVS to meet the country and global needs for health estimates at disaggregated levels. The 4th health sector programme for 2017–2021 identified 10 key driving forces for the next 5 years, including the ‘adoption of new technologies to strengthen surveillance, data quality and information systems to provide a strong evidence base for decision making’ [16].

Though sample surveys have been playing a key role in monitoring health sector progress in Bangladesh, there are some pressing issues with the use and interpretation of these data, e.g. reference periods for impact-level indicators will always provide slightly older estimates than the current status; and with gradual improvement of mortality levels, the surveys will increasingly require larger samples, which are costly. For example, given the current level of maternal mortality, the 2016 BMMS requires nearly 320,000 households to be surveyed for estimating a nationally representative maternal mortality ratio and it will quite possibly be the last BMMS for Bangladesh as future surveys will need to be considerably larger and more expensive. On the other hand, the routine HIS currently covers only the public sector and has issues with data quality. Also, the HIS can capture service utilization but not the actual demand for the services. In order to address these challenges, strengthening SVRS and other systems (e.g. strategically located sentinel surveillance systems to produce nationally representative data) could be a solution. With targeted investment and technical assistance, a strong SVRS of the national statistical agency (BBS) can be developed to facilitate monitoring of health outcomes at national and sub-national levels. In order to address fragmentation and non-existent private sector linkages, key services and mortality reporting can also be institutionalized at the facility as well as the community levels. We also agree to strategies proposed by Bryce and colleagues, which include linking household surveys to other sources of information on service provision [30].

CRVS, if properly functioning, could be a vital source of accurate data for health estimates. The GoB’s CRVS system is currently quite weak. A number of ministries including the Prime Minister’s Office are working through a Committee housed in the Cabinet Division for the implementation of CRVS. Much of their work is implemented through the Birth and Death Registration Project of the Local Government Division. An Office of Registrar General has very recently been established with the expectation to eventually take over the entirety of the CRVS operations. Since the HIS is uniquely positioned to assist in timely registration of births and deaths, effective linkages are being developed with the CRVS, including the electronic transmission of birth and death information.

As an effort to encourage different programmes to use a common information system, the MISs of DGHS and DGFP with technical support from MEASURE Evaluation, icddr,b, Save the Children, and other partners have been piloting a business automation process of the routine HIS to integrate parallel systems into a common platform like DHIS-2 (Bangladesh is the largest user of the DHIS-2 platform). This routine health information system (RHIS) strengthening initiative, primarily funded by USAID, is currently working in 2 districts on establishing an electronic H.I.S, with plans to expand to over 10 districts in the next year. Paper-based data recording and reporting tools are being replaced with applications on tablets, laptops, and desktops in the hands of government field and health facility workers. Under this initiative, a population registration system, based on a full census of the catchment area, has been developed for client tracking. When scaled up, the system has the potential to resolve the majority of the systemic challenges discussed here in generating high-quality, disaggregated data for monitoring the health sector’s progress. It is to be noted that capacity building of government health workers is crucial to rolling out such innovative systems.

Under the M&E Strategy for the health sector, the MOHFW outlined a Capacity Building Plan accompanied by time-bound action items to be implemented over five years. The Capacity Building Plan primarily focuses on facilitating and promoting M&E knowledge, skills, and competence within the MOHFW. It entails development of standardized M&E training materials and a multi-year training-of-trainers (TOT) programme for developing a cadre of skilled M&E trainers. The MIS units of MOHFW will continuously develop their capacity and remain up-to-date on recent international developments in M&E and in training methodologies. This needs to go together with improvements in data collection systems. In order to resolve data multiplicity, triangulation, and consistency issues, we propose the formation of an independent platform (tentatively titled the Bangladesh reproductive, maternal, newborn and child health (RMNCH) Indicator Reference Group – BRiRG) to play a crucial role. With appropriate technical capacity, the BRiRG will discuss and review data sources and findings, and undertake analysis with the intent to (a) provide clear and sound evidence on levels and causes of maternal, neonatal, and child morbidity and mortality in Bangladesh, and coverage of key interventions; (b) advise the GoB and other implementation partners on appropriate methods and assumptions for national and sub-national estimates; (c) help identify the most effective interventions and assess the impact of programmes delivering these interventions; and (d) build country capacity on data triangulation and issues with different estimation processes.

#### Recommended key actions

Rigorous efforts and strong political commitment are necessary for strengthening the production and use of estimates in Bangladesh’s health sector. Based on the situation analysis presented in this paper, we recommend the following key priorities to be undertaken to meet post-2015 data requirements:

- Fully implement the action plan outlined in the M&E Strategy and increase investments to move towards a fully digitized HIS.
- Assess and put in place the HR required for strengthening M&E systems.
- Develop and implement a multi-year, comprehensive capacity-building plan for improving M&E knowledge, skills, and competence in routine data collection, analysis, feedback, and use.
- Establish strict health data standards for all health facilities (public, private, and NGO sectors) to ensure cross-departmental (among different government agencies of the MOHFW) and cross-sectoral coordination (among different ministries), so that data are compatible.
- Enforce DQA mechanisms by integrating DQA procedures into the existing HIS with systematic verification procedures.
- Enhance demand for data, by establishing partnerships among agencies within the MOHFW and between the MOHFW and other ministries, research organizations, development partners, NGOs, and professional bodies – establishing an independent Indicator Reference Group can facilitate data analysis and support for evidence-based decision making.
- Explore opportunities to establish an oversight committee to develop standards, policies, guidelines, and protocols for data interoperability and ensuring privacy.

Bangladesh has made remarkable progress in strengthening its HIS. The country has been recognized with the United Nations Digital Health for Digital Development Award and was made an honorary sponsor of the Measurement and Accountability for Results in Health (MA4Health) Summit 2015 for performance in its HIS and e-health. Still, there is a long way to go in terms of achieving a sustainable digital HIS. Improved systems will improve data quality, availability, and use, and better support policymakers, with real-time data, to make informed decisions, and ultimately improve the health of the people of Bangladesh.

## Paper context

The data capturing system in Bangladesh’s health sector has undergone significant reforms in recent years. This paper critically analyzes the country situation in regard to production and use of health estimates from national sources, and the role of modeled estimates from international sources. The paper makes a strong case for investing more in routine health information systems, and building country capacity to resolve long-standing data limitations for progressing towards the post-2015 development agenda.
